# Calculation and analysis of the efficiency of resource allocation for technological innovation in China

**DOI:** 10.1371/journal.pone.0308960

**Published:** 2024-08-23

**Authors:** Ruying Chen, Lanyu Wu

**Affiliations:** School of Business, Linyi University, Linyi, Shandong, China; Middle Tennessee State University Jennings A Jones College of Business, UNITED STATES OF AMERICA

## Abstract

The efficiency of resource allocation in technological innovation is a critical factor influencing the output level of technological innovation. By expanding and optimizing the Hsieh & Klenow (2009) framework for analyzing the efficiency of resource allocation and relaxing the assumption of constant returns to scale, this study utilizes sample data from Chinese listed companies from 2007 to 2019 to measure and analyze the resource allocation efficiency level in China’s technological innovation. The findings indicate that in the process of technological innovation, companies face heterogeneous resource usage costs, leading to a deviation from the optimal resource allocation state, with evident issues of resource misallocation. The loss of efficiency in technological innovation output due to resource misallocation is significant, and addressing this issue can substantially enhance the level of technological innovation output. The misallocation of research and development capital resources is more severe than that of research and development personnel, resulting in greater efficiency losses in technological innovation output. Government subsidies are identified as a significant factor affecting resource allocation in technological innovation. Addressing the issue of resource misallocation, accelerating the market-oriented reforms of technological innovation resource allocation, and optimizing the government subsidy screening mechanism are crucial for improving the efficiency of resource allocation in technological innovation.

## Introduction

Technological innovation is recognized as the primary driver of technological progress and a central force propelling economic development [1–4]. Especially in the current phase of economic development, where economic growth is at a critical juncture of adjustment and transformation, it is particularly important to continue improving the level of technological innovation output. The Chinese government, adhering to an innovation-driven development strategy, has significantly increased investment in technological innovation resources. As reported by the World Bank, China’s R&D expenditure relative to GDP stood at 2.18% in 2018, comparable to the European Union’s average and nearing the developed countries’ average of 2.38%. It is undeniable that the substantial increase in investment in technological innovation resources is itself an important factor in the long-term rapid growth of technological innovation outputs. However, research suggests that the level of technological innovation output is more dependent on the efficiency of these innovations rather than the sheer scale of investment [5, 6]. Furthermore, the efficiency of resource allocation is increasingly viewed as a crucial element of output efficiency. Notably, the marketization of factor resources in China is still inadequate compared to product markets. Due to factors such as inappropriate local government subsidies and financing constraints, the efficiency of resource allocation in China is far below the level of developed countries. Serious resource misallocation issues and associated output efficiency losses cannot be ignored [7].

In this backdrop, accurately measuring and analyzing the efficiency of resource allocation in China’s technological innovation is fundamental for enhancing the output level from an efficiency standpoint. Therefore, this paper leverages and extends the Hsieh & Klenow framework [8] to examine various aspects: the presence of price distortions during enterprise investments in technological innovation resources, the efficiency of resource allocation within China’s technological innovation, the allocation efficiency of R&D personnel versus R&D capital, and their respective impacts on output. It also evaluates the influence of government subsidies on both technological innovation output and resource allocation in China.

This paper contributes in several ways: Firstly, it explores methods to augment China’s technological innovation output through the lens of micro-enterprise resource allocation, revealing actual resource allocation efficiency levels and the extent of losses in output efficiency. This provides a factual and theoretical basis for policy formulation. Secondly, it empirically assesses how government subsidies impact the efficiency of resource allocation in technological innovation, enhancing the understanding of the relationship between subsidies and innovation output. This analysis aids in refining and optimizing government subsidy programs. Lastly, by incorporating variable returns to scale (VRS) instead of constant returns to scale (CRS), this study offers a more realistic depiction of economic conditions, aiding in reducing estimation and measurement inaccuracies.

## Literature review

Improving the level of technological innovation output is an important means to enhance the efficiency and quality of economic output and plays a significant role in the sustainable and green economic development process [9–12]. The question of how to increase the level of technological innovation output has attracted widespread attention [13]. The process of technological innovation is essentially an input-output process [14, 15], thus, the pathway to enhance the level of technological innovation output can be achieved by increasing the intensity of technological innovation resource input and by enhancing the efficiency of technological innovation output [16–18].

Regarding the intensity of technological innovation resource input, studies have indicated that due to substantial external risks and other challenges encountered during the innovation process, there often exists severe market failure, resulting in investment levels in technological innovation that are far below the optimum [19]. Thus, increasing the intensity of technology resource input is a critical means to enhance the level of technological innovation output [20]. Compared to developed countries, the internal and external environments faced by the main bodies of scientific and technological innovation in developing countries are more adverse, with more severe market failure issues [21]. Therefore, through government policy subsidies, innovations in the financing system, and other mechanisms to address market failures, the level of scientific and technological innovation outputs can be effectively improved [22, 23].

Regarding the efficiency of technological innovation output, an increasing number of studies suggest that improvements in technological innovation capabilities stem not only from the continuous growth in the intensity of technological innovation inputs but also from the enhancement of technological innovation output efficiency [24–26]. Some studies believe that although China’s investment in technological innovation has increased significantly year by year, the efficiency of technological innovation output has not seen a marked improvement. To elevate the level of technological innovation output, more emphasis should be placed on the efficiency of technological innovation output [27]. There is substantial room for improvement in China’s technological innovation output efficiency. Simply increasing the "quantity" of technological innovation resource inputs while neglecting the "quality" of output efficiency improvement will result in a significant waste of technological innovation resources and tepid growth in innovation efficiency [28].

Regarding resource allocation efficiency, studies have found that when factor price distortions exist, resource allocation efficiency is generally lower than what would be optimally expected, and resource misallocation can lead to significant losses in output efficiency [29–31]. From a research perspective, factor resource allocation can be distinguished into meso-level and micro-enterprise-level studies [32]. Meso-level studies on resource misallocation suggest that the actual allocation of resources among industries or regions deviates significantly from optimal allocation, resulting in output efficiency losses [33, 34]. Micro-enterprise-level studies indicate that the core issue of factor resource misallocation occurs at the micro-enterprise level, where the output efficiency losses due to resource misallocation are considerable. Improvements in resource allocation efficiency at this level could greatly enhance overall output efficiency [7, 35–37]. Current research on resource allocation efficiency is mainly focused on the production field [38–40], with few scholars paying attention to the resource allocation efficiency in the field of technological innovation. It is posited that there is a significant misallocation of technological innovation resources among regions or industries in China, which is a major factor contributing to the low levels of technological output. Thus, improving the efficiency of technological innovation resource allocation is deemed urgent [41, 42].

In summary, the significance of technological innovation in contemporary economic development is increasingly acknowledged. Research aimed at enhancing technological innovation has primarily focused on reducing innovation failures and boosting inputs for technological innovation, often overlooking the influence of resource allocation efficiency on technological innovation output levels. Moreover, while existing studies on the efficiency of technological innovation resource allocation are predominantly concentrated at regional or industry levels, they tend to overlook the efficiency of resource allocation among micro-enterprises. Thus, drawing from the aforementioned research gaps, this paper applies and expands the prevalent micro-level resource misallocation analysis framework to assess and analyze the efficiency of resource allocation in technological innovation. Building on this framework, it further empirically tests the effects of government subsidy policies on the level of technological innovation output and the state of resource allocation.

## Theoretical analysis

### Basic setup

By referencing and expanding the theoretical analysis framework of Hsieh and Klenow, this study constructs a three-tier production decision model. This model relaxes the assumption of constant returns to scale and compares the input and output levels of micro-enterprises in the technological innovation process under different resource allocation efficiency states. The theoretical analysis explores the causes of technological innovation resource misallocation and its impact on the efficiency of technological innovation output. It is assumed that the final output of technological innovation is in a perfectly competitive market with representative firms, and the form of their technological innovation function is as follows:

$$Y={∐}_{s=1}^{S}Y_{s}^{\theta_{s}}$$ (1)

In Eq (1), $\sum_{s=1}^{S}\theta_{s}=1$; *Y* represents the final output of technological innovation; *Y*_*s*_ denotes the technological innovation output of industry *s*, serving as the resource input for the final output of technological innovation; and *θ*_*s*_ represents the output elasticity of intermediate input *Y*_*S*_ in technological innovation.

It is assumed that the form of the technological innovation function for the technological innovation output of industry *s* is as follows:

$$Y_{s}={\left(\sum_{i=1}^{M_{s}}Y_{si}^{\frac{\sigma -1}{\sigma }}\right)}^{\frac{\sigma }{\sigma -1}}$$ (2)

In Eq (2), *Y*_*si*_ represents the technological innovation output of enterprise *i* within the industry, and *σ* represents the substitution elasticity between *Y*_*si*_.

Furthermore, it is assumed that enterprise *i* faces a monopolistically competitive market in the process of technological innovation, and its output *Y*_*si*_ technological innovation function adopts the Cobb-Douglas (C-D) form:

$$Y_{si}=A_{si}K_{si}^{\alpha_{s}}L_{si}^{\beta_{s}}$$ (3)

In Eq (3), *A*_*si*_ represents the technological innovation output efficiency of enterprise *i*, similar in meaning to the total factor productivity during the micro-enterprise production process; *K*_*si*_ and *L*_*si*_ respectively represent the input quantities of R&D capital and R&D personnel during the technological innovation process; *α*_*s*_ and *β*_*s*_ respectively represent the output elasticities of R&D capital and R&D personnel in technological innovation, assuming that the output elasticities of technological innovation resources for all enterprises within industry *s* are the same. To reveal the impact of returns to scale on the allocation of technological innovation resources and to more accurately measure the efficiency of technological innovation resource allocation, this section, unlike the studies of Hsieh and Klenow (2009) and others, no longer confines itself to the assumption of constant returns to scale.

### Analysis of resource misallocation efficiency

Assuming that enterprises face resource price distortions during the technological innovation process, the price distortion coefficients for R&D personnel and R&D capital in technological innovation are defined as *τ*_*K*_ and *τ*_*L*_ respectively. For instance, when an enterprise faces higher costs of capital usage and labor, *τ*_*K*_ and *τ*_*L*_ will increase; conversely, they will decrease. Under these conditions, the expression for maximizing enterprise technological innovation benefits becomes:

$$\underset{{K_{si},L}_{si}}{max}P_{si}Y_{si}-(1+\tau_{L_{si}})p_{L}L_{si}-(1+\tau_{K_{si}})p_{K}K_{si}$$ (4)

In Eq (4), *p*_*K*_ and *p*_*L*_ respectively represent the theoretical usage prices of R&D capital and R&D personnel, which, in the absence of price distortions, are also equal to the actual usage prices; *K*_*si*_ and *L*_*si*_ respectively represent the input quantities of R&D capital and R&D personnel; *P*_*si*_ represents the price of enterprise technological innovation output.

Using Eq (2) and the first-order condition for industry profit maximization, the relationship between *P*_*si*_ and *Y*_si_ can be calculated as (where *P*_*s*_ represents the price of industry technological innovation output):

$$P_{si=}P_{s}{Y_{s}}^{\frac{1}{\sigma }}{Y_{si}}^{-\frac{1}{\sigma }}$$ (5)

Under resource price distortions, combining Eqs (4) and (5), through the first-order conditions for profit maximization, we obtain the expressions for the quantities of R&D personnel and R&D capital:

$$K_{si}\propto {\left(1+\tau_{K_{si}}\right)}^{-1}{\left[(1+\tau_{K_{si}}{)}^{\alpha_{s}}{\left(1+\tau_{L_{si}}\right)}^{\beta_{s}}{A_{si}}^{-1}\right]}^{(1-\sigma )H_{s}}$$ (6)

$$L_{si}\propto {\left(1+\tau_{L_{si}}\right)}^{-1}{\left[(1+\tau_{K_{si}}{)}^{\alpha_{s}}{\left(1+\tau_{L_{si}}\right)}^{\beta_{s}}{A_{si}}^{-1}\right]}^{(1-\sigma )H_{s}}$$ (7)

Combining Eqs (3), (6), and (7), we derive the level of R&D output:

$$Y_{si}\propto {\left[A_{si}(1+\tau_{K_{si}}{)}^{-\alpha_{s}}{\left(1+\tau_{L_{si}}\right)}^{-\beta_{s}}\right]}^{\sigma H_{s}}$$ (8)

In Eqs (6)–(8), $H_{s}=1/\left[\left(\alpha_{s}+\beta_{s})-\sigma (\alpha_{s}+\beta_{s}-1\right)\right]$. Eqs (6)–(8) demonstrate that when resource price distortions exist, the R&D capital, R&D personnel, and technological innovation output in the enterprise’s technological innovation process are all affected by factor price distortions, deviating from the optimal inputs and outputs where no price distortions exist. At this time, the efficiency of resource allocation among enterprises is lower than the optimal allocation, indicating the presence of resource misallocation. Consequently, it is possible to determine whether resource misallocation exists by measuring the heterogeneity in the prices of technological innovation resources used among enterprises. If enterprises face the same resource usage prices, i.e., the marginal outputs of resources are equal, then resources are optimally allocated among enterprises, and there is no resource misallocation problem; if enterprises face heterogeneous resource usage prices, i.e., the marginal outputs of resources are unequal, then resources will deviate from the optimal allocation among enterprises, indicating a resource misallocation problem. Further, Eqs (6)–(8) show that when returns to scale are variable, i.e., when *H*_*s*_ is not equal to 1, the extent to which factor price distortions affect resource allocation is moderated by variable returns to scale. This moderation through variable returns to scale can amplify or reduce the impact of factor price distortions on factor resource allocation. Therefore, imposing the strict condition of constant returns to scale, when actual returns to scale are variable, will lead to two main issues: on one hand, it will cause bias in the estimation of factor output elasticity; on the other hand, it will overlook the moderating effect of changes in returns to scale on factor resource allocation. As a result, these two aspects will lead to inaccuracies in measuring resource allocation efficiency.

On the basis of enterprise technological innovation output efficiency A_si_, combining Eqs (3) and (4), the enterprise’s technological innovation income output efficiency is defined as:

$$TFPR_{si}=P_{si}A_{si}=\frac{\sigma }{\sigma -1}{\left(\frac{MRPL_{si}}{\alpha_{s}}\right)}^{\alpha_{s}}{\left(\frac{MRPK_{si}}{\beta_{s}}\right)}^{\beta_{s}}$$ (9)

In Eq (9), *MRPK*_*si*_ and *MRPL*_*si*_ respectively represent the marginal outputs of the enterprise’s technological innovation R&D capital and R&D personnel. Their expressions are derived from the first-order condition of Eq (4) and are given by $p_{K}(1+\tau_{K_{si}})\;and\;p_{L}(1+\tau_{L_{si}})$, respectively. Eq (9) indicates that if there is no factor price distortion, enterprises will face the same resource usage prices, and their resource marginal outputs will be equal, then the levels of enterprise technological innovation income output efficiency are also equal. At this time, resources are optimally allocated among enterprises, and there is no resource misallocation problem; conversely, the opposite is true.

Based on the aforementioned equations, the expression for industry technological innovation output efficiency is further derived as:

$$TFP_{s}={\left[{\sum_{i=1}^{M_{s}}\left(A_{si}{{\left(\frac{MRPK_{s}}{MRPK_{si}}\right)}^{\alpha_{s}}(\frac{MRPL_{s}}{MRPL_{si}})}^{\beta_{s}}{\left(\frac{P_{s}Y_{s}}{P_{si}Y_{si}}\right)}^{1-(\alpha_{s}+\beta_{s})}\right)}^{\sigma -1}\right]}^{\frac{1}{\sigma -1}}$$ (10)

In Eq (10), *MRPK*_*s*_ and *MRPL*_*s*_ respectively represent the marginal outputs of the industry’s technological innovation R&D capital and R&D personnel. Specifically, these are equal to the factor prices divided by the industry’s distortion coefficients, which are aggregated from the enterprise distortion coefficients weighted by the proportion of each enterprise’s output value. Further derived is the expression for resource allocation efficiency, which is the ratio of the actual output efficiency level to the optimal output efficiency level:

$$EFF={∐}_{s=1}^{S}{\left(\frac{TFP_{s}}{TFP_{se}}\right)}^{\theta_{s}}$$ (11)

Here, *TFP*_*se*_ represents the optimal efficiency level of industry technological innovation output when there are no factor price distortions. Similarly, to compare the degrees of resource misallocation for R&D capital and R&D personnel, the expressions for resource allocation efficiency considering only one of the two factors can be derived as:

$${EFF}_{K}={∐}_{s=1}^{S}{\left(\frac{TFP_{s}}{TFP_{seK}}\right)}^{\theta_{s}}$$ (12)

$${EFF}_{L}={∐}_{s=1}^{S}{\left(\frac{TFP_{s}}{TFP_{seL}}\right)}^{\theta_{s}}$$ (13)

Where *TFP*_*seK*_ and *TFP*_*seL*_ respectively represent the optimal efficiency levels of industry technological innovation output when there are no distortions in the prices of R&D capital and R&D personnel.

## Parameter estimation method and data description

### Parameter estimation method

The theoretical analysis framework indicates that a prerequisite for calculating the efficiency of technological innovation resource allocation is to clarify the output elasticity coefficients of industry technological innovation resources. This paper posits that, similar to the production process, the enterprise technological innovation process also faces simultaneity selection bias, which directly results in the underestimation of the output elasticity coefficient of R&D capital. To overcome simultaneity selection bias, this paper adopts the OP method [43] to estimate the resource output elasticity coefficients. The OP method assumes that firms make investment decisions based on their current productivity levels. Therefore, the firm’s current investment can be used as a proxy variable for unobservable productivity shocks, addressing the issue of simultaneity bias. The empirical estimation model is established as follows:

$$LnY_{it}=\alpha +\beta_{1}LnK_{it}+\beta_{2}LnL_{it}+\beta_{3}Age_{it}+\beta_{4}State_{it}$$

$$+\sum \lambda_{m}year_{m}+\sum \lambda_{n}ind_{n}+\sum \lambda_{s}pro_{s}+\varepsilon_{it}$$ (14)

In Eq (14), LnY represents the logarithmic value of the quantity of enterprise technological innovation output; LnK and LnL respectively represent the logarithmic values of the quantity of enterprise R&D personnel and R&D capital; Age represents the age of the enterprise; State is a dummy variable indicating whether the enterprise is state-owned, coded as 1 for state-owned enterprises and 0 otherwise; year is a dummy variable for the year; ind is a dummy variable for the industry; pro is a dummy variable for the region. When using the OP method, the state variables(state) are LnK and Age; the control variable (cvars) is State; the proxy variable(proxy) is LnI, measured by the logarithm of R&D investment; and the exit variable(exit) is Exit, which is generated based on the firm’s survival and operational status; other variables such as LnL, year, ind and pro are treated as free variables(free).

### Data description

As this paper uses micro-enterprise sample data, the main databases available for selection are the China Listed Firms Database and the China Industrial Enterprises Database. Due to the absence of key indicators such as R&D personnel, R&D capital, and technological innovation output in the China Industrial Enterprises Database, this paper selects data from A-share listed enterprises in China as the empirical research sample, with a sample interval from 2007 to 2019. To ensure the quality of empirical research, financial industry listed companies, ST companies, *ST companies, and samples missing key indicators are excluded.

The definitions of the main variables are as follows:

Enterprise technological innovation output(Y) is represented by the number of patent authorized.

Enterprise R&D capital(K) is calculated using the perpetual inventory method, with the formula: $K_{t}={(1-\delta )K}_{t-1}+I$, where δ represents the depreciation rate, set at 15% following the setting in mainstream literature, and I represents R&D invest.

Enterprise R&D personnel(L) is represented by RD personnel.

The price of R&D personnel is represented by the average wage of urban unit employees in scientific research, technical services, and geological exploration.

The price of R&D capital follows general principles is represented by a 10% capital rental price.

The substitution elasticity between enterprise technological innovation outputs is set at 3, following the practice in literature such as Hsieh and Klenow.

The statistical description of the main variables is shown in Table 1.

**Table 1:** The statistical description of the main variables.

Variable Name	Sample Size	Mean	Standard Deviation	Minimum Value	Maximum Value
LnY	11,401	2.82	1.43	0.00	9.50
LnK	11,401	19.21	1.37	10.34	26.23
LnL	11,401	5.92	1.19	1.39	11.50
LnI	11,401	17.86	1.33	7.17	23.47
Age	11,401	16.36	5.60	2.00	52.00
State	11,401	0.29	0.46	0.00	1.00
Exit	11,401	0.22	0.42	0.00	1.00

### Calculation results and analysis

Using the OP method, the output elasticity coefficients of industry technological innovation resources were estimated. To minimize the impact of extreme values in micro samples on the estimation results, a 1% double-sided truncation was applied to the main variables during the estimation process. In addition, to verify the accuracy of the coefficient estimation results, a comparative analysis was conducted using the Ordinary Least Squares (OLS) method, the Fixed Effects (FE) method, and the Constrained Ordinary Least Squares (OLS) method, where the sum of the estimated output elasticity coefficients for R&D capital and R&D personnel is constrained to equal 1.The empirical results are presented in Table 2.

**Table 2:** Parameter estimation results.

Variable	OLS Method	FE Method	Constrained OLS Method	OP Method
LnK	0.283*** (26.91)	0.273*** (13.56)	0.379*** (29.95)	0.578*** (6.12)
LnL	0.439*** (37.03)	0.341*** (16.55)	0.621*** (49.07)	0.280*** (13.89)
Year Dummy Variable	YES	YES	YES	YES
Region Dummy Variable	YES	YES	YES	YES
Industry Dummy Variable	YES	YES	YES	YES
N	11401	11401	11401	11401
R-sq	0.464	0.521	0.426	

^a^ Numbers in parentheses are standard errors. N represents the number of samples. R-sq indicates the R-squared value.

* P<0.1

** P<0.05

*** P<0.01.

First, a comparison of the results in Table 2 indicates that the estimated output elasticity coefficients of both resources are significant at the 1% level under all four estimation methods. The output elasticity coefficient of R&D capital obtained using the OP method is higher than those obtained using the other three methods, while the output elasticity coefficient of R&D personnel is lower. This implies that using the OP method to estimate the factor marginal output coefficients is appropriate. The OP method effectively addresses simultaneity selection bias and sample selection bias, enhancing the contribution level of R&D capital and more accurately estimating the output elasticity coefficients of the two factors.

Furthermore, the sum of the estimated output elasticity coefficients for R&D capital and R&D personnel is less than 1 under the OLS, FE, and OP methods, indicating variable returns to scale in the process of technological innovation. Relaxing the assumption of constant returns to scale aligns with the actual conditions of technological innovation output. Under the assumption of variable returns to scale, subsequent estimates, such as the factor price distortion coefficient, factor marginal output coefficient, and resource allocation efficiency, will be more accurate. This helps in understanding and grasping the true efficiency of resource allocation in the process of technological innovation. In contrast, the Constrained Ordinary Least Squares (OLS) method constrains the sum of the estimated output elasticity coefficients for R&D capital and R&D personnel to equal 1, deviating from the reality of variable returns to scale. Additionally, the estimated output elasticity coefficients for R&D capital (0.379) and R&D personnel (0.621) using the Constrained OLS method are significantly different from those obtained using the other methods. This method seems to overstate the contribution of R&D personnel while understating the contribution of R&D capital. This is particularly evident when compared to the OP method, which is considered relatively accurate, where the estimated output elasticity coefficients for R&D capital and R&D personnel are 0.578 and 0.280, respectively.

Finally, the results from the OP method indicate that the output elasticity coefficient of R&D capital is greater than that of R&D personnel, suggesting that in the process of technological innovation, the contribution of R&D capital to output is more significant than that of R&D personnel.

Theoretical analysis reveals that factor price distortions in the process of enterprise technological innovation are a prerequisite for resource misallocation. Therefore, based on the estimated output elasticity coefficients of R&D capital and R&D personnel, further calculations of resource price distortion coefficients are carried out. It is important to note that since the theoretical prices of resources are set artificially, there may be a discrepancy between them and their actual values. Although this discrepancy does not affect the calculated value of resource allocation efficiency, it does influence the value of the resource price distortion coefficient for individual enterprises. Therefore, the existence of price distortions in resource usage cannot be determined solely by whether the resource price distortion coefficient equals 1. The accurate method of determination is to verify whether the resource price distortion coefficients for all enterprises are equal. If they are equal, it indicates that all enterprises face the same resource usage prices, and there is no misallocation of technological innovation resources among enterprises. Conversely, if they are not equal, it indicates that the prices of resources used by each enterprise differ, and there is a misallocation of technological innovation resources among enterprises. Furthermore, the greater the difference in the resource price distortion coefficients among enterprises, the more severe the misallocation of technological innovation resources; conversely, the smaller the difference, the less severe the misallocation. To eliminate the impact of different dimensions and compare the degree of variation in resource price distortions for R&D capital and R&D personnel, the coefficient of variation for the resource price distortion coefficients of R&D capital and R&D personnel is calculated.

First, the results in Table 3 indicate that, under both constant and variable returns to scale assumptions, the coefficients of variation for the resource price distortion coefficients of R&D capital and R&D personnel are significantly non-zero each year. This suggests that there are evident differences in the prices enterprises pay for technological resources, indicating a significant presence of misallocation in technological innovation resources. Second, the variation in resource price distortion for R&D capital is greater than that for R&D personnel, indicating a more severe misallocation issue for R&D capital resources. Finally, although the coefficients of variation for the resource price distortion coefficients of R&D capital and R&D personnel are similar under both constant and variable returns to scale assumptions, there are significant differences in the estimated output elasticity coefficients of R&D capital and R&D personnel under these two assumptions. Because the output elasticity coefficients of innovation resources deviate from their true values under the assumption of constant returns to scale, the impact of resource misallocation on technological innovation output will also deviate from the actual situation.

**Table 3:** Results of the coefficient of variation for factor price distortion coefficients.

Year	τ_K_		τ_L_	
	CRS	VRS	CRS	VRS
2007	1.48	1.48	1.46	1.46
2008	1.63	1.58	1.72	1.71
2009	1.67	1.68	1.42	1.43
2010	1.30	1.28	1.21	1.21
2011	1.37	1.39	1.12	1.19
2012	1.35	1.33	1.21	1.21
2013	1.27	1.28	1.13	1.14
2014	1.38	1.36	1.12	1.12
2015	1.32	1.29	1.12	1.12
2016	1.35	1.33	1.10	1.11
2017	1.28	1.29	1.07	1.08
2018	1.24	1.18	1.05	1.04
2019	1.23	1.20	1.08	1.09
Average	1.37	1.36	1.22	1.22

Note: CRS and VRS represent constant returns to scale and variable returns to scale, respectively.

Through the aforementioned analysis, the existence and degree of misallocation of the two types of resource in the technological innovation process can be preliminarily determined. Based on the aforementioned calculation and analysis, further calculations are made for the efficiency value of technological innovation output when there is misallocation of technological innovation resources, as well as the potential improvement value of technological innovation output efficiency. The calculation results are shown in Table 4. The results indicate that the loss in efficiency of technological innovation output caused by misallocation of technological innovation resources is very severe. On average, due to resource misallocation, the actual efficiency of technological innovation output is only about 55.58% of the optimal output efficiency, with an average potential improvement value of 82.39%. In other words, if the problem of misallocation of technological innovation resources were completely eliminated, and assuming the quantity of resources invested in technological innovation remains unchanged, the output of technological innovation would increase by 82.39%. Although in reality, due to factors like information asymmetry and the cost of factor mobility, it is difficult to completely eliminate resource misallocation. However, it can be imagined that if the efficiency of China’s technological innovation resource allocation could be improved, the level of technological innovation output would also see a significant increase.

**Table 4:** Results of technological innovation resource allocation efficiency (Unit: %).

Year	EFF	TFP_G	EFF_K_	TFP_GK	EFF_L_	TFP_GL
2007	72.66	37.62	78.77	26.95	93.55	6.89
2008	59.18	68.98	75.08	33.20	82.96	20.54
2009	59.41	68.33	71.82	39.23	86.99	14.96
2010	55.42	80.44	67.28	48.64	87.10	14.81
2011	51.32	94.85	64.36	55.38	86.90	15.08
2012	47.43	110.84	60.46	65.41	85.25	17.30
2013	50.74	97.07	63.42	57.68	86.37	15.78
2014	51.80	93.03	64.90	54.08	86.80	15.21
2015	47.41	110.93	60.31	65.81	86.85	15.14
2016	51.33	94.82	64.33	55.45	86.59	15.49
2017	54.19	84.54	67.82	47.46	87.25	14.61
2018	57.79	73.05	71.05	40.75	90.14	10.94
2019	63.85	56.63	76.01	31.57	90.50	10.50
Average	55.58	82.39	68.12	47.82	87.48	14.40

Note: TFP_G = (1/Eff) - 1, represents the potential improvement in the efficiency of technological innovation output when resource misallocation is completely eliminated. The expressions for TFP_GK and TFP_GL are similar to that of TFP_G, and they respectively represent the potential improvement in the efficiency of technological innovation output when the misallocation of R&D capital or R&D personnel resources is completely eliminated.

Differentiating by factors, consistent with the calculated results and analysis above, the loss in technological innovation output efficiency caused by misallocation of R&D capital resources is relatively more severe. The actual efficiency of technological innovation output due to misallocation of R&D capital resources averages 68.12% of the optimal output efficiency, with an average potential improvement value of 47.82%. This indicates that although China’s investment intensity in technological innovation remains at a certain level, the investment in technological innovation R&D funds has not been fully allocated according to the efficiency of enterprise technological innovation output. Some enterprises with high technological innovation output efficiency have not reached the optimal level of investment in technological innovation R&D funds; some enterprises with low technological innovation output efficiency have exceeded the optimal level of investment, leading to a suboptimal allocation of existing R&D capital resources and a technological innovation output level far below the optimal.

The misallocation of R&D capital resources can be attributed to several factors. One major reason is the inefficient distribution of government subsidies. While these subsidies are intended to support technological innovation, hey often are not allocated based on the actual efficiency of firms’ innovation outputs. Another significant factor is market imperfections, such as information asymmetry. Additionally, inconsistent policy implementation contributes to this issue. Addressing these challenges through more efficient subsidy distribution, improved access to information and financial resources, and consistent policy implementation can significantly enhance the allocation efficiency of R&D capital and boost technological innovation output.

The average actual efficiency of technological innovation output, due to the misallocation of R&D personnel resources, is 87.48% of the optimal output efficiency, with an average potential improvement value of 14.40%. Clearly, the problem of misallocation of R&D personnel resources is not as severe as that of R&D capital resources. Consequently, the loss in technological innovation output efficiency it causes is also relatively minor, indicating that the allocation of R&D personnel resources has already reached a relatively high level.

When analyzed annually, the efficiency of technological innovation resource allocation exhibits a trend of initially declining, then rising. The initial decline can likely be attributed to the impact of the international financial crisis, during which national economic growth rates slowed, and most enterprises faced more severe internal and external operating environments. Coupled with the high risk associated with technological innovation activities, some enterprises might have restricted their investment in technological innovation elements to alleviate temporary operational difficulties, thus exacerbating the problem of technological innovation resource misallocation. During the subsequent rising phase, the likely reasons include the implementation of policies such as ’Made in China 2025’ and supply-side structural reforms. These initiatives have led to breakthroughs in the process and mechanisms for market-oriented allocation of technological factors. Additionally, enhancements in the government’s guidance and service capabilities have improved the efficiency of technological innovation resource allocation. This analysis also indirectly indicates that the level of China’s technological innovation resource allocation efficiency is far below what it should be. If the roles of the market and government are further utilized, the efficiency of technological innovation resource allocation will continue to improve, leading to significant growth in technological innovation output.

To facilitate comparison with the assumption of constant returns to scale, we conducted calculations of resource allocation efficiency under this assumption. The results, presented in Table 5, indicate that while the trend in resource allocation efficiency measurements under constant returns to scale generally aligns with those observed under variable returns to scale, the resource allocation efficiency coefficient calculated under constant returns to scale is generally lower. Consequently, the assumption of constant returns to scale tends to overestimate the degree of resource misallocation in technological innovation.

**Table 5:** Comparison of resource allocation efficiency measurement results under the assumptions of constant returns to scale and variable returns to scale (Unit: %).

Year	EFF		TFP_G	
	CRS	VRS	CRS	VRS
2007	62.67	72.66	59.56	37.62
2008	45.00	59.18	122.21	68.98
2009	51.97	59.41	92.42	68.33
2010	49.90	55.42	100.42	80.44
2011	47.14	51.32	112.13	94.85
2012	41.25	47.43	142.40	110.84
2013	46.91	50.74	113.18	97.07
2014	45.19	51.80	121.27	93.03
2015	43.75	47.41	128.57	110.93
2016	46.02	51.33	117.28	94.82
2017	47.56	54.19	110.24	84.54
2018	51.02	57.79	96.00	73.05
2019	57.77	63.85	73.11	56.63
Average	48.94	55.58	106.83	82.39

Note: CRS and VRS represent constant returns to scale and variable returns to scale, respectively.

To verify the accuracy of the resource allocation efficiency calculation results mentioned above, this study conducts validation in different aspects:

Treatment of Extreme Values: Initially, the study employed a 1% double-sided trimming of extreme values. It now shifts to a 2% double-sided trimming. This adjustment is expected to reduce the fluctuation range of resource allocation efficiency results while maintaining the overall trend, if the calculation method is accurate and reliable.

**Fig 1:**
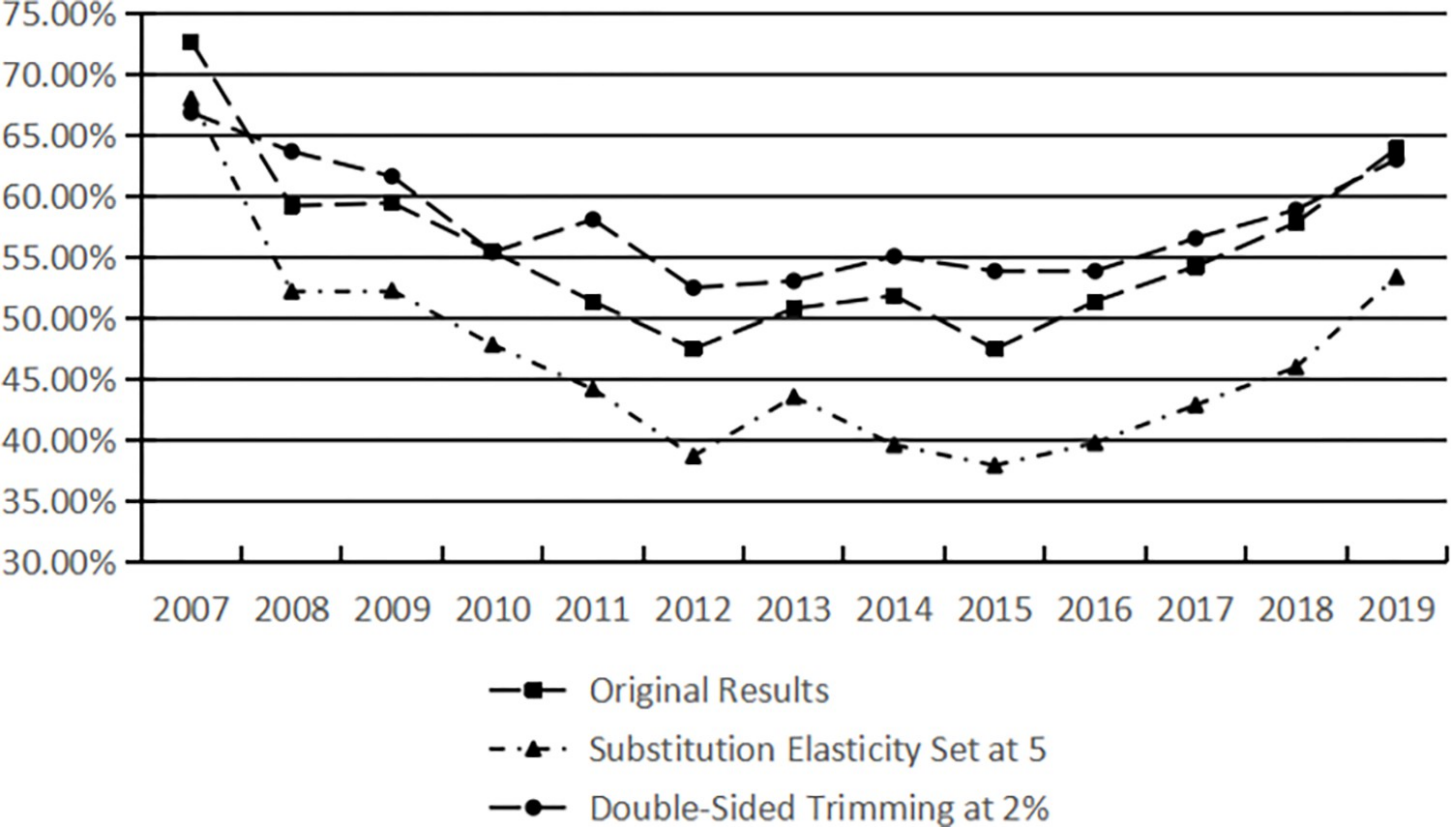
Calculation results of resource allocation efficiency under different conditions.

Setting of Substitution Elasticity between Enterprises’ Technological Innovation Outputs: The substitution elasticity value shifts from 3 to 5. To facilitate a comparison with the original calculation results, the results under these new conditions are displayed graphically in Fig 1. The results indicate that, after applying a 2% double-sided trimming, the calculated values for resource allocation efficiency are marginally higher than the original results, yet with a few exceptions, the overall trend remains consistent. Similarly, setting the substitution elasticity for enterprises’ technological innovation outputs to 5 results in slightly higher efficiency calculations than the original, but the overall trend still aligns closely with previous findings. These observations confirm the robustness and reliability of the original calculations in this study.

## Further analysis

Globally, government industrial policies have been widely applied as a key means to promote economic development [44, 45]. Government subsidies, as an important component of industrial policies, theoretically play roles in reducing externalities, correcting market failures, and optimizing resource allocation [46–48]. The impact of government subsidies on technological innovation has received widespread attention, with many scholars agreeing that government subsidies positively influence innovation by alleviating financing constraints and encouraging investment in innovation [49–54]. However, existing research has often overlooked the impact of government subsidies on the efficiency of resource allocation for technological innovation. In light of this, this paper aims to empirically examine the effect of government subsidies on technological innovation and further empirically verify the impact of government subsidies on the allocation efficiency of resources for technological innovation.

To test the impact of government subsidies on enterprise technological innovation output levels, the following empirical test model is established:

$${LnY}_{it}=\alpha +\beta LnSub_{it}+\delta X_{it}+\sum \lambda_{m}year_{m}+\sum \lambda_{n}ind_{n}+\sum \lambda_{s}{pro}_{s}+\varepsilon_{it}$$ (15)

In Eq (15), the dependent variable LnY is defined as in Eq (14). The variable LnSub represents the logarithm of the government subsidy amount, serving as a measure of the intensity of government subsidies. The control variables X include: LnK, LnL, Scale, State and Age. Here, Scale is the enterprise scale indicator, categorized by the quartile division of total enterprise assets. The definitions for the other control variables remain consistent with those in Eq (14).

To verify the impact of government subsidies on the state of allocation of technological innovation resources, the following empirical model is established:

$${Cv}_{it}=\alpha +\beta LnSub_{it}+{\delta X}_{it}+\sum \lambda_{m}year_{m}+\sum \lambda_{n}ind_{n}+\sum \lambda_{s}{pro}_{s}+\varepsilon_{it}$$ (16)

In Eq (16), the variable Cv encompasses two distinct variables, CvK and CvI, defined by the discrepancies between the firm-specific values τ_K_ and τ_L_ and their respective industry averages. These differences aim to assess the effectiveness of government assistance in optimizing the allocation of R&D capital and labor resources during the technological innovation process. The control variables X include: Scale, State and Age.

Employing the Fixed Effects (FE) model, we empirically assessed the influence of government subsidies on enterprise technological innovation output, with findings presented in Table 6. As anticipated, across various model settings, the coefficients of government subsidies were significantly positive, demonstrating a robust promotional effect on enterprise technological innovation output, aligning with established research. Given potential endogeneity issues from reverse causality, which could lead to biased estimates, we selected the first-order lag of government subsidies as an instrumental variable. A two-stage least squares (2SLS) method was then applied to ensure robust estimation. To validate the choice of the instrumental variable, we conducted tests confirming its relevance to the endogenous explanatory variable and its independence from the disturbance term. The unidentifiability test employs the Kleibergen-Paap rk LM method, and the weak instrumental variable test employs the Cragg-Donald Wald F statistic method, judged by the critical values provided by Stock and Yogo [55]. The test results show that the selected instrumental variable does not exhibit any issues with unidentifiability or weakness as an instrumental variable, and the choice is rational. The 2SLS results show that the impact coefficient of government subsidies is significant and consistent with the FE fixed effects model method, further confirming the robustness and reliability of the empirical results.

**Table 6:** Impact results of government subsidies on enterprise technological innovation output level.

Variable	FE Method		2SLS Method
Lnsub	0.0457*** (0.0108)	0.0410*** (0.0111)	0.236** (0.111)
LnK	0.317*** (0.0309)	0.295*** (0.0301)	0.307*** (0.0506)
LnL	0.210*** (0.0285)	0.194*** (0.0272)	0.171*** (0.0347)
Scale	0.135*** (0.0247)	0.146*** (0.0231)	0.0773* (0.0461)
State	-0.300** (0.130)	-0.172 (0.121)	-0.309** (0.127)
Age	0.0467*** (0.00791)	0.0587*** (0.0119)	0.0194** (0.00878)
_cons	-6.260*** (0.510)	-4.689*** (0.558)	
Year Dummy Variable	NO	YES	NO
Region Dummy Variable	NO	YES	NO
Industry Dummy Variable	NO	YES	NO
Kleibergen-Paap rk LM_P			0.0000
Cragg-Donald Wald F			71.193
N	9990	9990	7177
R-sq	0.279	0.316	0.172

^a^ Numbers in parentheses are standard errors. N represents the number of samples. R-sq indicates the R-squared value

* P<0.1

** P<0.05

*** P<0.01.

The Fixed Effects (FE) model was employed to empirically assess the impact of government subsidies on the allocation of R&D capital and personnel resources, with findings detailed in Table 7. The analysis reveals that government subsidies significantly influence the allocation of R&D capital resources, implying that such subsidies are pivotal in altering the state of R&D capital resource distribution and significantly affect its allocation efficiency. This insight enhances our understanding of how government subsidies influence technological innovation levels, indicating that subsidies not only enhance technological innovation at the enterprise level but also modify the distribution of R&D capital among firms. Conversely, the allocation of R&D personnel resources appears unaffected by government subsidies.

**Table 7:** Results of the impact of government subsidies on enterprise technological innovation resource allocation.

Variable	CvK		CvI	
Lnsub	0.0995*** (0.0383)	0.114*** (0.0422)	0.0163 (0.0208)	0.0201 (0.0208)
Scale	0.105 (0.0819)	0.120 (0.0810)	0.0164 (0.0499)	-0.00622 (0.0463)
State	-0.421* (0.232)	-0.409* (0.224)	-0.110 (0.186)	-0.00819 (0.189)
Age	-0.115*** (0.0204)	-0.267*** (0.0805)	-0.0388*** (0.0125)	-0.0739*** (0.0255)
_cons	0.910* (0.547)	3.097* (1.590)	0.809** (0.357)	2.806*** (0.697)
Year Dummy Variable	NO	YES	NO	YES
Region Dummy Variable	NO	YES	NO	YES
Industry Dummy Variable	NO	YES	NO	YES
N	9990	9990	9990	9990
R-sq	0.012	0.051	0.004	0.043

^a^ Numbers in parentheses are standard errors. N represents the number of samples. R-sq indicates the R-squared value

* P<0.1

** P<0.05

*** P<0.01.

In robustness test, we utilized the ratio of government subsidy amounts to R&D investment amounts as a proxy variable for the intensity of government subsidies to evaluate their impact on the allocation of technological innovation resources. The results, detailed in Table 8, corroborate the preliminary findings, demonstrating that government subsidies significantly affect the allocation of R&D capital resources, while their impact on the allocation of R&D personnel resources remains insignificant.

**Table 8:** Robustness test results.

Variable	CvK		CvI	
Sub	0.0870*** (0.00561)	0.0496*** (0.00630)	-0.00341 (0.00261)	-0.00290 (0.00270)
Scale	-0.0690** (0.0283)	0.160*** (0.0535)	0.0195 (0.0490)	-0.00181 (0.0457)
State	0.0916 (0.0694)	-0.452 (0.289)	-0.116 (0.186)	-0.0166 (0.189)
Age	0.0127** (0.00582)	-0.241*** (0.0243)	-0.0367*** (0.0125)	-0.0703*** (0.0259)
_cons	2.546*** (0.335)	4.491 (2.829)	1.042*** (0.165)	3.103*** (0.602)
Year Dummy Variable	NO	YES	NO	YES
Region Dummy Variable	NO	YES	NO	YES
Industry Dummy Variable	NO	YES	NO	YES
N	10067	10067	10067	10067
R-sq	0.030	0.056	0.004	0.042

^a^ Numbers in parentheses are standard errors. N represents the number of samples. R-sq indicates the R-squared value

* P<0.1

** P<0.05

*** P<0.01.

## Conclusions and policy implications

This paper employs the micro-level resource allocation efficiency analysis framework of Hsieh and Klenow, and relaxes the assumption of constant returns to scale. It employs data from Chinese listed companies spanning 2007 to 2019 to calculate and analyze the efficiency of resource allocation for technological innovation in China. The primary conclusions are as follows:

Theoretical Analysis: The analysis indicates that when enterprises encounter heterogeneous resource usage prices during the technological innovation process, the quantities of inputs and outputs deviate from their optimal levels. This deviation signifies the presence of resource misallocation, where the actual efficiency of technological innovation output fails to achieve optimal output efficiency, leading to diminished efficiency. Importantly, these losses are moderated by variable returns to scale.

Empirical Analysis: The results find that Chinese enterprises face significant price distortions when utilizing technological innovation resources, leading to considerable efficiency losses attributable to resource misallocation. Addressing this misallocation could substantially improve the level of technological innovation output. Further, the analysis highlights that the misallocation of R&D capital resources is more severe than that of R&D personnel resources. Given the higher contribution of R&D capital to technological innovation output, the inefficiency in technological innovation output due to the misallocation of R&D capital resources is notably more pronounced. Moreover, government subsidies play a crucial role in enhancing the level of enterprise technological innovation output. These subsidies also significantly influence the allocation of technological innovation resources, indicating their vital role in supporting and directing the innovation capacities of enterprises.

Based on the above conclusions, the policy implications are:

Addressing the Issue of Technological Innovation Resource Allocation: The state of resource allocation is a crucial factor affecting the output of technological innovation. Close attention to issues of resource misallocation is essential for a deep understanding of the development process of technological innovation and for exploring the drivers of future innovation outputs. Therefore, in analyzing issues related to technological innovation output, the impact of inefficient resource allocation must not be overlooked.

Advancing Marketization Reform of Technological Innovation Resources: It is essential to address the financing constraints that hamper enterprise technological innovation. The misallocation of R&D capital resources constitutes a significant aspect of overall technological innovation resource misallocation. To mitigate this, it is critical to further the marketization of financial capital. This includes strengthening the fundamental role of market mechanisms in allocating financial resources, enhancing the financing structure, and reducing information asymmetry through the adoption of innovative mechanisms and systems. Such reforms are crucial for effectively enhancing the availability of funds necessary for enterprise technological innovation.

Enhance the Government Subsidy Screening Mechanism: Government subsidy policies play a pivotal role in alleviating market failures in technological innovation by directing resources towards enterprises with high innovation efficiency. It is imperative to optimize the subsidy screening mechanism to ensure that these subsidies are effectively enhancing resource allocation efficiency and, consequently, the level of technological innovation output. Such optimization should focus on refining efficiency selection mechanisms within the government’s subsidy allocation process, guiding subsidy resources toward enterprises that demonstrate high innovation efficiency.

These recommendations are highly practical, particularly in refining the government subsidy screening mechanism to ensure that resources are more effectively directed towards the most innovative enterprises. However, several potential implementation challenges must be addressed. First, accurately assessing the innovation efficiency of enterprises necessitates the establishment of a detailed and dynamic evaluation system, which could demand significant time and resources. Second, issues such as data transparency and information asymmetry could complicate the acquisition of accurate data on enterprise innovation. Third, the consistency and stability of government policies are crucial; frequent policy changes might disrupt enterprises’ expectations and their reliance on government subsidies. Therefore, it is crucial to comprehensively consider these challenges and develop corresponding strategies to ensure the effective implementation of these recommendations.

## Supporting information

S1 Data The dataset used in this article for discussion and analysis. (XLSX)
